# Effects of Non-periodized and Linear Periodized Combined Exercise Training on Insulin Resistance Indicators in Adults with Obesity: A Randomized Controlled Trial

**DOI:** 10.1186/s40798-021-00359-x

**Published:** 2021-09-26

**Authors:** Anne Ribeiro Streb, Larissa dos Santos Leonel, Rodrigo Sudatti Delevatti, Cláudia Regina Cavaglieri, Giovani Firpo Del Duca

**Affiliations:** 1 Department of Physical Education, Research Center in Physical Activity and Health, Federal University of Santa Catarina, Florianópolis, Santa Catarina Brazil; 2 Exercise Physiology Laboratory - FISEX, Faculty of Physical Education, University of Campinas (UNICAMP), Campinas, Brazil

**Keywords:** Chronic disease, Insulin resistance, Adult, Exercise, Clinical Trial

## Abstract

**Background:**

The aim was to verify the effect of non-periodized and linear periodized combined (aerobic plus resistance) exercise training on insulin resistance markers in adults with obesity.

**Methods:**

A blinded randomized control trial was conducted with three groups of individuals with obesity (BMI, 30–39.9 kg/m^2^): control group (CG, *n* = 23), non-periodized group (NG, *n* = 23), and linear periodized group (PG, *n* = 23). The NG and PG performed aerobic and resistance exercises in the same session in aerobic-resistance order for 16 weeks. Both intervention groups trained three sessions weekly, with a total duration of 60 min each. The aerobic training of the NG had a duration of 30 min always between 50% and 59% of the reserve heart rate (HRres), while resistance exercise was comprised of 6 exercises, performed always in 2 × 10–12 maximum repetitions (MRs). The PG progressed the aerobic and resistance training from 40%–49% to 60%–69% (HRres) and from 2 × 12–14 to 2 × 8–10 RM, respectively, along the intervention period. The evaluated indicators of insulin resistance included fasting glucose, fasting insulin, and homeostasis model assessment-estimated insulin resistance (HOMA-IR) collected pre- and post-intervention. The analyses to verify the exercise training effect were performed using generalized estimating equations.

**Results:**

After 16 weeks of training, per protocol analysis (*n* = 39) showed significant reductions in HOMA-IR only in the training groups (NG: *Δ* **=** − 1.6, PG: *Δ* **=** − 0.6; *p* = 0.094). Intention-to-treat analysis demonstrated significant reductions in fasting insulin levels (NG: *Δ* **=** − 1.4, PG: *Δ* **=** − 1.0; *p* = 0.004) and HOMA-IR (NG: *Δ* **=** − 5.5, PG: *Δ* **=** − 3.8; *p* = 0.002).

**Conclusion:**

Periodized and non-periodized combined exercise training similarly reduces insulin resistance markers in adults with obesity.

*Trial registration*: Brazilian Registry of Clinical Trials, RBR-3c7rt3. Registered 07 February 2019—https://ensaiosclinicos.gov.br/trial/5970/1.

## Key Points

- Combined exercise training promotes significant reductions in insulin resistance indicators in adults with obesity.
- Periodized and non-periodized combined exercise training similarly reduces insulin resistance markers.
- Starting training at low or moderate intensity promotes similar results in inactive adults with obesity.
- Even with low weekly frequency, combined physical exercise improves glycemic metabolism.
- Adults with obesity have low adherence to treatment with physical exercise.

## Background

Obesity is considered a global health problem affecting 13% of the world population [1], with estimates indicating that 18.9% of the Brazilian population has this disease [2]. Excessive accumulation of ectopic fat is associated with metabolic disorders. The adipose tissue hypertrophy alters adipokine profiles to promote a pro-inflammatory profile, which favors insulin resistance [3] and greatly increases the chances of developing type 2 diabetes mellitus(T2DM) [4]. Indeed, the term "diabesity" has been used in view of the close relationship between these diseases [5]. Corroborating this, individuals with obesity tend to have higher insulin resistance values, with a higher incidence of dysglycemia, hypertriglyceridemia, and high blood pressure [6, 7]. Moreover, even individuals considered to have metabolic healthy obesity are more likely to develop other comorbidities and are more prone to all-cause mortality [8].

Regarding glycemic metabolism in individuals with obesity, an important strategy to prevent the occurrence of T2DM and associated complications is the practice of exercise training, which is an effective and low-cost tool, recommended worldwide [9]. Among the types of training, it has been observed that a combination of aerobic and resistance exercises, called combined training, generates a sum of benefits from both modalities, and is recommended for health promotion and longevity of adults with and without obesity and diabetes [10, 11]. Acutely, a combined training session has superior results in β-cell function, insulin sensitivity, and glucose levels compared to aerobic or resistance training alone [12], also, these benefits can be extended or consonant chronically with such practice [13, 14].

However, changes resulting from training are subject to strategies that aim to modulate training variables such as intensity, volume, recovery interval, and exercise order in programs; thus, periodization is employed for this purpose [15–17]. The use of periodization is related to general health benefits for the population with obesity [18–20]. Progressive overload, planned recovery, and the variety inherent to periodization promote gains in physical fitness. A monotonous training, with little or no variation, can induce psychological symptoms [20], such as mood disturbances, depression, apathy, and lack of motivation, frequently related to the population with obesity. On the other hand, the gradual progression of intensity can increase adherence [21], as well as the self-efficacy [22]. It is known that adherence to exercise is related to the results obtained (dose–response), including indicators of insulin resistance [23]. Though, regarding the improvement of glucose metabolism in this population, several models have been used and, therefore, it is difficult to interpret. Additionally, comparisons are often made using isolated aerobic or resistance exercise [15, 17] rather than combined training, which is the widely recommended modality for the management of obesity.

However, improvements in glycemic metabolism resulting from different combined training models are frequently investigated with T2DM population, and further understanding of the effect of periodic combined training in the population with obesity is required due to the early presentation of significant changes in glucose metabolism, especially insulin resistance, which may collaborate to trigger diabetes mellitus. Moreover, the effects of the periodization on clinical outcomes in special populations, such as individuals with obesity, are still emergent. Tracking cases of obesity, which commonly precede multimorbidity, and identifying training strategies that improve or even maintain indicators of glycemic metabolism may provide insights for the treatment and prevention of such chronic diseases.

To fill the gap and create a pioneer study, we aimed to verify the effect of non-periodized and linear periodized combined exercise training on insulin resistance markers in adults with obesity. We hypothesized that both training models would benefit insulin resistance markers but that training combined with linear periodization would be superior to non-periodized training.

## Methods

### Study Design

This study was a blind randomized controlled trial that included three groups of individuals with obesity, conducted in parallel over 16 weeks. The present study is part of a larger project, entitled “Effects of different protocols of adult health training on people with obesity”, which was approved by the Ethics Committee and Research on Human Beings of the institution of origin (protocol 2.448.674) and registered in the Brazilian Registry of Clinical Trials (RBR-3c7rt3). Furthermore, this study was performed in with the standards of ethics outlined in the Declaration of Helsinki. The methodological details of the larger project of this study are described in its protocol [24].

### Participants

The initial disclosure of the study was made in electronic and printed media. Interested volunteers contacted the researchers via an online form to be filled in order to verify their eligibility. The eligibility criteria included: age between 20 and 50 years, grade I or II obesity in terms of body mass index (BMI) (30–34.9 kg/m^2^ and 35–39.9 kg/m^2^, respectively)[25], and no physical exercise with a weekly frequency of more than twice a week in the past 3 months. In addition, participants could not present with any cardiometabolic disease and/or use continuous medications, as well as not using medications to control and/or treat obesity, nor having performed any surgical procedure aimed at weight reduction. Volunteers who were previously considered eligible were called for a face-to-face interview, where pre-participation health screening was carried out. Those who met all the criteria and consented to participate were included in the study and provided written informed consent.

### Randomization

All participants underwent a series of evaluations before being randomly allocated to three groups: control group (CG), non-periodized training group (NG), and linear periodization group (PG). Randomization was stratified by sex, age, and BMI, collected at baseline, with a ratio of 1:1:1 by the site www.randomizer.com. This process was conducted by independent researchers who were not involved in the evaluations or the intervention process. The allocation list was only unveiled to trainers on the start date of the intervention.

### Interventions

The NG and PG participated in 16 weeks of combined training (including aerobic and resistance exercises in the same session). Aerobic training was performed continuously through walking and/or running, with intensities prescribed by percent reserve heart rate ranges (%HRres). Resistance training was performed in multiple sets, using six exercises, all performed with weight training equipment, with prescription for maximal repetition ranges. The increase in load (kilograms) was indicated whenever the participants sustained the expected series in the upper repetition range for two consecutive sessions.

**Fig. 1:**
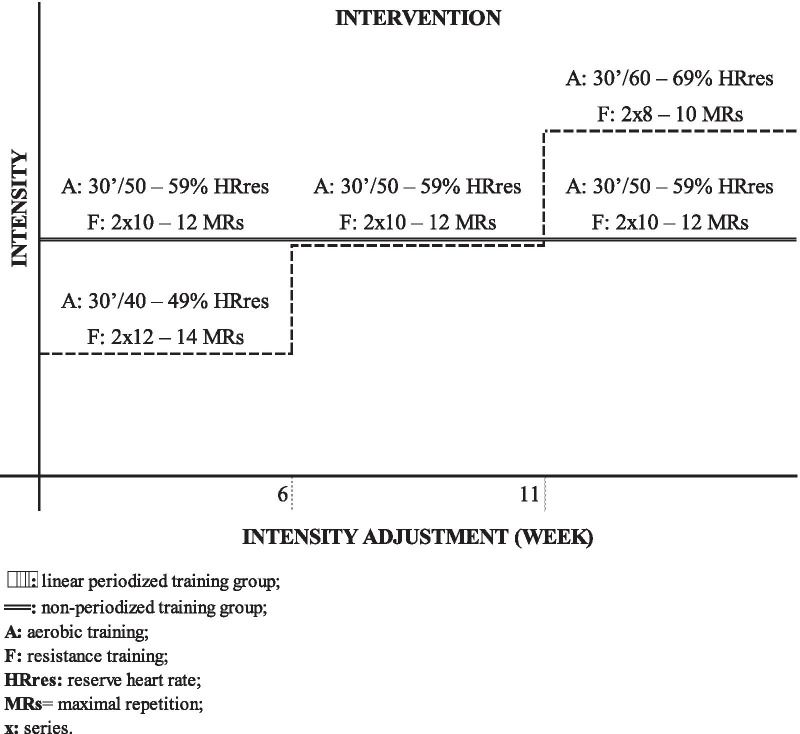
Structure of the training protocols performed by the periodized combined training group (PG) and the non-periodized training group (NG)

The established weekly frequency was three times, with an average duration of 60 min; the first 5 min for warm-up, 30 min for aerobic training, 20 min for resistance training, and the final 5 min for stretching. The first week was used as training familiarization for both groups. Afterward, the PG participated in training with increasing linear periodization, which was divided into three mesocycles of five weeks each (40%–49%, 50%–59%, and 60%–69% [HRres] and 2 × 12–14, 2 × 10–12 to 2 × 8–10 [RM], respectively), while the NG remained at moderate intensity (50–59% HRres and 2 × 10–12 RM) throughout the study (Fig. 1). In the end, the training volume of both groups was equivalent. The CG did not receive any intervention and the participants were instructed to maintain routine activities. Additional methodological details can be found in the study protocol [24].

### Assessment for Sample Characterization and Exercise Prescription

Participants completed an online questionnaire on the Question Pro Platform, containing sociodemographic information. For the body composition evaluation, a tetrapolar bioelectrical impedance InBody 720 (Ottoboni, Rio de Janeiro, Brazil) was used and manipulated by experienced evaluators who followed the recommended protocol to use the equipment [26]. To prescribe physical training, the maximum and resting heart rates were used to calculate the ideal training zone; these were obtained using Polar® portable heart rate monitors, model S810i. The maximum heart rate was measured by exercise test until voluntary treadmill exhaustion (Imbramed Millenium Super ATL, Porto Alegre, Brazil), according to the protocol previously validated by Jones and Doust [27]. The resting heart rate was recorded with the participant lying down with a heart rate monitor strap positioned. Three notes were taken over 5 min (minutes 3, 4, and 5), and the average was recorded as a reference value. Resting heart rate was reassessed at the end of each mesocycle to adjust intensity.

### Outcome Assessments

Outcomes were obtained by venipuncture, where 20 ml samples were collected in dry bottles with separating gel and another in parallel with anticoagulant (EDTA). Collections took place between 7 and 9 a.m., and the participants fasted for 12 h before collection. Post-treatment collections were made between 48 and 72 h after the last exercise session. Blood samples were processed and centrifuged to obtain plasma and serum, before storing in a -80ºC bio freezer. The evaluated indicators of insulin resistance were fasting glucose, fasting insulin, and insulin resistance (HOMA-IR). An enzymatic-colorimetric kit (Trinder) was used according to the manufacturer's recommendations for the determination of fasting glucose values. The serum insulin concentration values in mU/L were measured by chemiluminescence immunoassay using the ADVIA CentaurXP ™ Automated Chemiluminescence System. Both analyses were performed at the Clinical Analysis Laboratory of the University Hospital of the Federal University of Santa Catarina. Insulin resistance was estimated using the insulin resistance homeostasis model (HOMA-IR) using the formula: HOMA-IR = [fasting glucose (mmol/L) * fasting insulin (uU/ml)]/22.5.

### Statistical Analysis

The sociodemographic variables, sex (male or female), marital status (with and without a partner), skin color (white or black), and schooling (high school and college) were used to characterize the sample. Continuous variables were expressed as mean and standard deviation, and categorical variables were expressed as relative frequency and percentage. Data distribution was verified using the Shapiro–Wilk test. Differences between the groups at baseline were tested by one-way analysis of variance for independent samples (one-way ANOVA) and Chi-square test.

The sample size calculation was calculated a priori in the Gpower 3.1.9.2 software, considering the glucose variable. The stipulated effect size was 0.18, considering the error of type *α* = 0.05 and error of type *β* = 0.80. The sample size indicated was 78 participants. Outcomes were analyzed by per protocol analysis in those who participated until the end of the study and had complete post-evaluation data. Outcomes were also analyzed by intention-to-treat analysis, in which all randomized participants were included, and the missing values were imputed by regression predictive factors by the maximum likelihood estimator given by generalized estimating equations (GEE). Intra- and intergroup analyses were also performed by GEE with the adoption of the Bonferroni post hoc test. Data are expressed as mean and standard error. The level of significance adopted for the interaction was *p* < 0.10, while for the isolated effect of time and/or group was *p* < 0.05. Montgomery (2013) [28] suggests that to analyze the generalized interactions of factors, it can consider using p values greater than usual for determining a significant effect. Therefore, considering group and time factors in the interaction analysis, we chose to consider the p-value of 0.10 for interactions in this study. All analyses were performed using IBM SPSS version 21.0 (IBM Corp., Armonk, NY, USA). The intra-group effect sizes (ES) were calculated by Cohen's *d*-test [29]; for this, the value of dividing the mean difference between each intra-group assessment was considered by grouping the standard deviation between the same assessment period. According to Cohen (1988), it was agreed that *d* values are considered small if (0.20 ≤ *d* < 0.50), medium if (0.50 ≤ *d* < 0.80), and large if (*d* ≥ 0.80) [29].

For fasting glucose and insulin data, the descriptive results of the individual responses of the participants analyzed per protocol are presented.

## Results

**Fig. 2:**
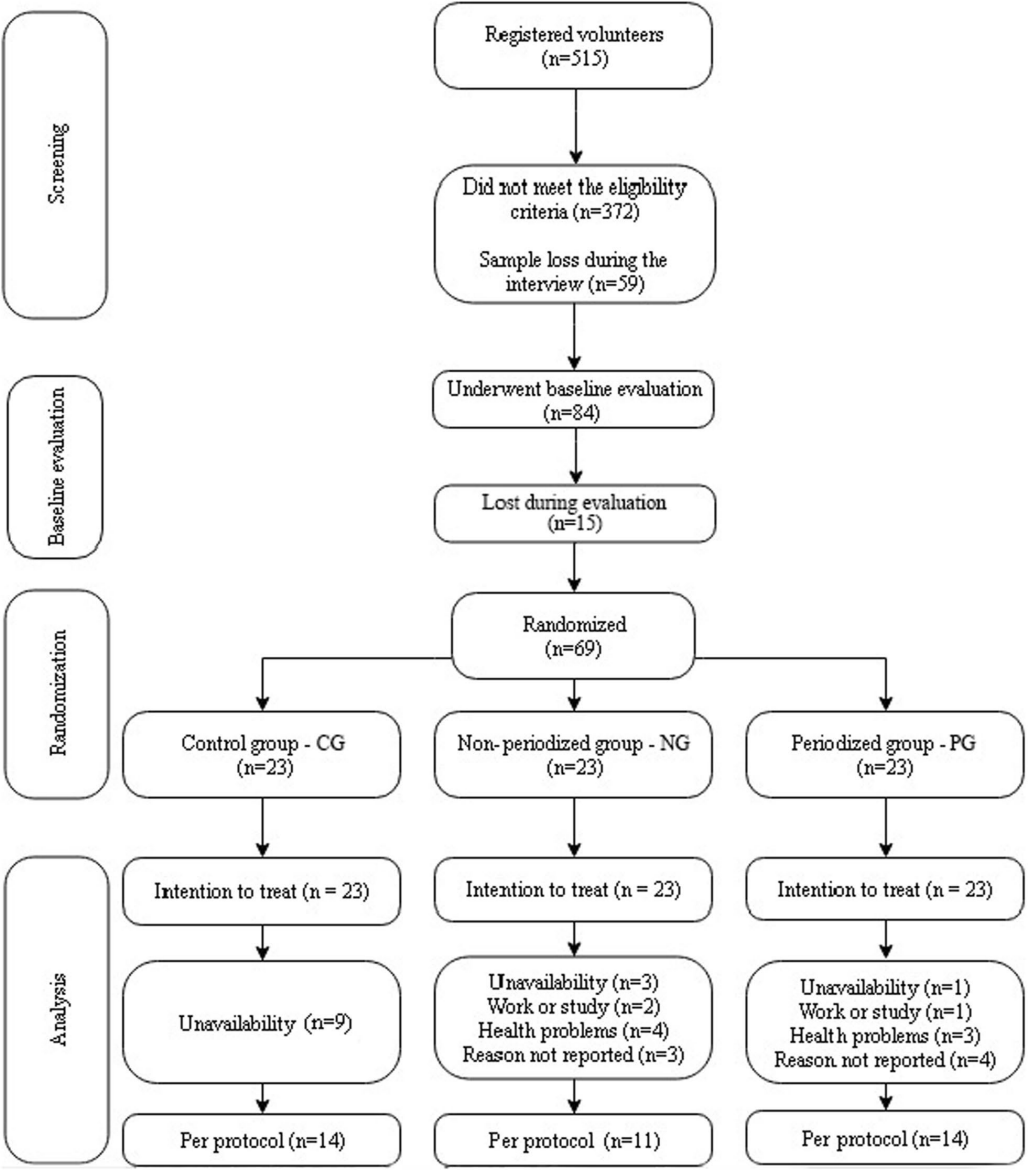
Study Flowchart

More than 500 volunteers applied for the study; however, after the initial evaluation processes, 69 participants remained and were randomized into three equal groups (NG, PG, and CG). After sixteen weeks of training, part of the sample was lost for reasons such as unavailability, work or study, and even health problems (Fig. 2).

A total of 39 people completed all phases of the trial and were included in the per-protocol analyses. The frequency of sessions was 64% and 61% for NG and PG, respectively, with no differences in aerobic (*p* = 0.350) and resistance training volume (*p* = 0.987). The overall average weekly frequency was 2.0 sessions in the first mesocycle, while in the third mesocycle it was 1.6, with no significant differences between training groups. However, the intensity proposed during the sessions was met by 90% of the participants. There were no adverse events during the exercises during the study.

Table 1 shows the baseline comparison of the sociodemographic characteristics and nutritional status of adults with obesity participating in the trial. The study sample had a mean BMI of 33.3 kg/m^2^ (± 3.13 kg/m^2^) and an age of 36 years (± 6 years). Most of the participants were women, who lived with a partner, with white skin color, and had completed high school.

**Table 1:** Characteristics of study participants (*n* = 69)

Variables	CG (23)	NG (23)	PG (23)	*p* value
	${\textbf{X}}$ (± sd)	${\textbf{X}}$ (± sd)	${\textbf{X}}$ (± sd)	
Age (years)	34.2 (± 7.6)	34.2 (± 6.7)	35.6 (± 7.4)	0.740
BMI (kg/m^2^)	33.2 (± 2.4)	33.7 (± 3.0)	33.5 (± 3.1)	0.129

	*n* (%)	*n* (%)	*n* (%)	
Sex				
Female	14 (60.9)	14 (60.9)	14 (60.9)	1.000
Marital status				
With a partner	17 (73.9)	14 (60.9)	13 (56.5)	0.442
Skin color				
White	19 (82.6)	19 (82.6)	18 (78.3)	0.910
Educational level				
High-school	18 (78.3)	18 (79.7)	18 (78.3)	0.914

*n* = absolute frequency; % = relative frequency

*X* = average; ± *dp* = standard deviation

*CG* control group, *NG* non-periodized group, *PG* periodized group, *BMI *body mass index

Table 2 shows the insulin resistance indicators with analysis per protocol and intention-to-treat. In the per protocol analysis, HOMA-IR was reduced in the training groups (NG- pre: 4.1 ± 0.9; post: 2.5 ± 0.4; PG- pre: 3.3 ± 0.3; post: 2.7 ± 0.4; *p* = 0.094) with medium effect size. The intention-to-treat analysis, despite not showing any significant difference, demonstrated that the fasting insulin (CG = 0.17; NG = 0.74; PG = 0.52) and HOMA-IR (CG = 0.12; NG = 0.84; PG = 0.60) decreased in all groups with medium and high effect size for training groups.

**Table 2:** Blood glucose, insulin and insulin resistance for control group (CG), non-periodized group (NG) and periodized group (PG) before and after 16 weeks of intervention

Group	Pré-intervention ${\textbf{X}}$ (± se)	Post-intervention ${\textbf{X}}$ (± se)	P-value				
			Mean difference	Cohen d	group	time	Group * time
Per protocol (*n* = 39)							
Blood glucose (mg/dL)							
CG (*n* = 14)	95.0 (± 1.0)	101.0 (± 4.0)	6.0	0.58	0.584	0.788	0.102
NG (*n* = 11)	97.1 (± 2.7)	91.9 (± 3.6)	− 5.2	0.54			
PG (*n* = 14)	97.0 (± 3.4)	94.7 (± 3.2)	− 2.3	0.20			
Insulin (mU/L)							
CG (*n* = 14)	15.3 (± 2.1)	16.1 (± 2.1)	0.8	0.11	0.438	0.072	0.141
NG (*n* = 11)	16.8 (± 3.4)	10.8 (± 1.6)	− 6.0	0.75			
PG (*n* = 14)	13.8 (± 1.2)	11.6 (± 1.5)	− 2.2	0.46			
HOMA-IR							
CG (*n* = 14)	3.6 (± 0.5)	4.0 (± 0.9)	0.4	0.15	0.441	0.130	0.094
NG (*n* = 11)	4.1 (± 0.9)	2.5 (± 0.4)*	− 1.6	0.76			
PG (*n* = 14)	3.3 (± 0.3)	2.7 (± 0.4)*	− 0.6	0.49			
By intention to treat (*n* = 69)							
Blood glucose (mg/dL)							
CG (*n* = 23)	99.8 (± 4.1)	99.6 (± 3.4)	− 0.2	0.01	0.426	0.124	0.614
NG (*n* = 23)	96.2 (± 1.8)	92.4 (± 3.1)	− 3.8	0.31			
PG (*n* = 23)	97.5 (± 2.1)	94.3 (± 3.1)	− 3.2	0.25			
Insulin(mU/L)							
CG (*n* = 23)	16.8 (± 2.1)	15.2 (± 1.8)	− 1.6	0.17	0.536	0.002	0.430
NG (*n* = 23)	16.4 (± 1.8)	10.9 (± 1.3)	− 5.5	0.74			
PG (*n* = 23)	16.0 (± 1.4)	12.2 (± 1.7)	− 3.8	0.52			
HOMA-IR							
CG (*n* = 23)	4.1 (± 0.5)	3.8 (± 0.5)	− 0.3	0.12	0.405	0.004	0.447
NG (*n* = 23)	3.9 (± 0.4)	2.5 (± 0.3)	− 1.4	0.84			
PG (*n* = 23)	3.9 (± 0.3)	2.9 (± 0.4)	− 1.0	0.60			

*X* = average; ± se = standard error

*CG* control group, *NG* non-periodized group, *PG* periodized group

Generalized estimated equation (GEE); Bonferroni post hoc test

*Significant difference intra-groups (*p* < 0.05)

**Fig. 3:**
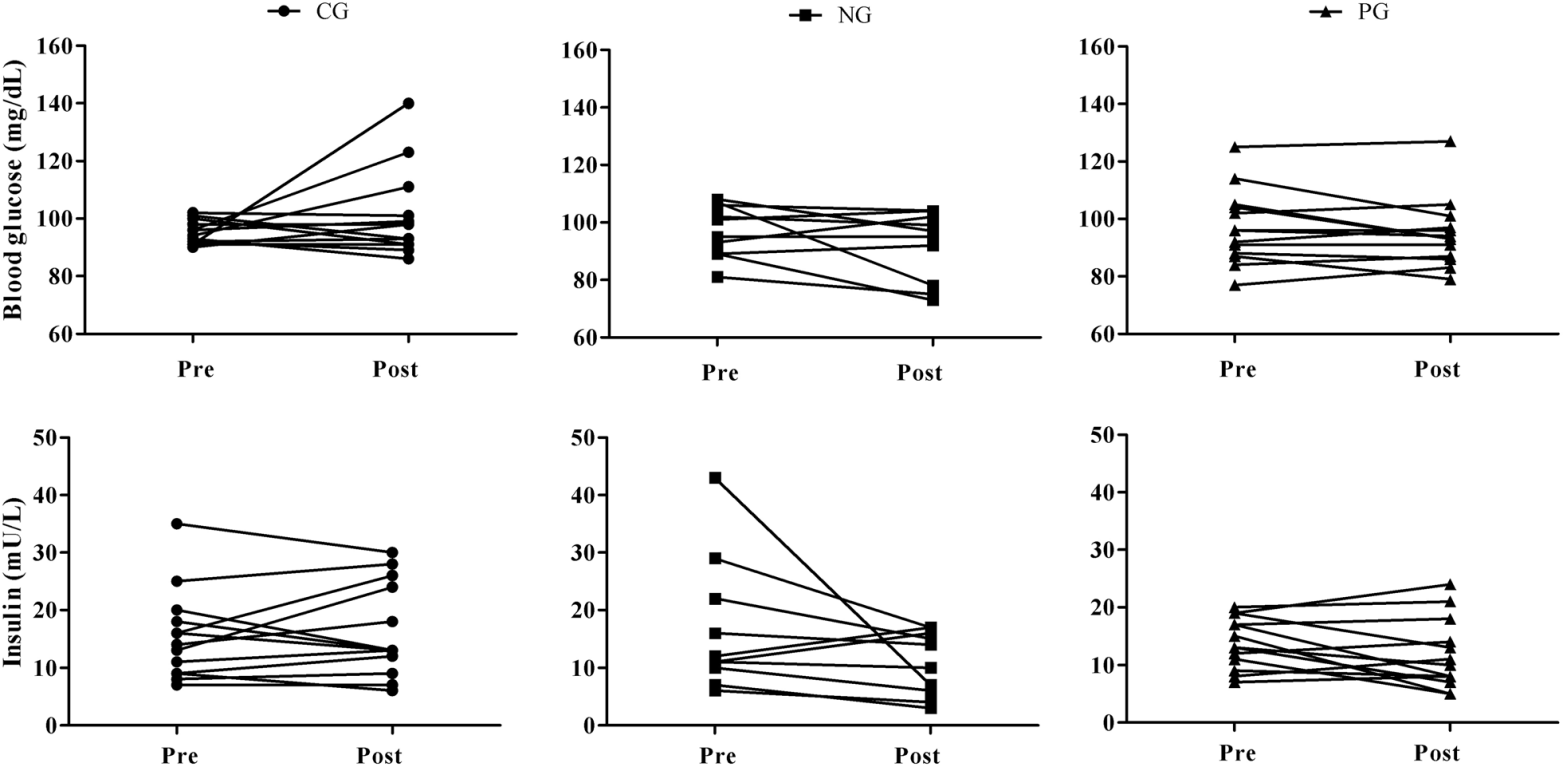
Individual responses of blood glucose and insulin according to the groups

Figure 3 shows the individual response data to fasting glucose and insulin according to the groups, respectively. This descriptive information allows the visualization of positive results predominantly in the groups that participated in the training, even without statistical significance.

## Discussion

In the current study, the aim was to determine the effect of 16 weeks of combined training with and without linear periodization on insulin resistance indicators in adults with obesity. The markers investigated included blood glucose, insulin, and HOMA-IR. Clinical improvements were observed for both training groups. In the per-protocol analysis for HOMA-IR, the interaction between group and time indicated equivalence of periodization types for the reduction of insulin resistance, refuting the initial hypothesis of superiority to the PG. The interpretation of this finding can be added to the result of another study published previously, using the same participants, where similar effects were reported in the increase in maximum strength of upper and lower limbs for both training groups, and the body composition remained unchanged [30]. It is possible that the muscular hypertrophy acquired with training causes endocrine modulations, mainly related to the increase in insulin sensitivity, regardless of the change in body composition [31].

Our findings corroborate those of Bonfante et al., (2017) that 24 weeks of combined training with linear periodization in men with obesity resulted in improvements in the aforementioned indicators[13]. These improvements in insulin markers in the population with obesity are important because are against of obesity pathophysiology, that is characterized by a state of low-grade systemic inflammation [32] due to increased secretion of inflammatory cytokines, such as TNF-α and IL-6. In addition, it increases the secretion of leptin, resistin, and inhibitor-1 of plasminogen activation, which promotes insulin resistance [33]. Physical exercise, in turn, moderates the inflammatory condition, decreasing the secretion of leptin and TNF-α, which is a metabolic cascade of changes in other adipokines, it reduces the secretion and cytokines of the insulin antagonist and subsequently improves insulin resistance [34]. Reductions in the indicators of such resistance are important and affect the improvement of this mechanism, which, in the long run, interferes with the appearance of the metabolic syndrome and T2DM [33].

Regarding fasting blood glucose, no significant changes were detected in our study, regardless of the group or analysis. Despite this, when analyzing only the participants who completed the training, it is possible to notice a decrease in fasting blood glucose. This finding can be partially explained because the serum values ​​are already normal at baseline, according to the reference values ​​established by the Brazilian Diabetes Society (2018) [35], and this minimizes the scope for improvement. However, these participants, who were already in a state of metabolic abnormality, were able to keep their glycemic levels within normal limits due to increased insulin secretion. This fact is relevant in this scenario, since physiological changes, such as increased pro-inflammatory cytokines and free circulating fatty acids, as well as reduced insulin sensitivity, increase the need for insulin secretion to maintain glycemic homeostasis. Over time, and with the worsening of the disease, hyperglycemia will establish itself due to the saturation of insulin production, as well as by resistance mechanisms [10]. It is important to emphasize that these variables were not measured by us. The literature presented is being used as support to support the data observed here. In addition, the evidence points to the importance of training volume and intensity for significant results of blood glucose [36, 37]. The present study, in turn, had a training protocol with moderate intensity and volume considered relatively low, which may also explain the findings in this variable.

In the per protocol analysis, HOMA-IR showed significant reductions over time only in the combined training groups. However, in the intention-to-treat analysis, in addition to HOMA-IR, fasting insulin also indicated reductions over time, with effect sizes of moderate magnitude for the intervention groups. Ahmadizad et al. (2014) and Inoue et al. (2015) investigated in their respective studies the effect of dissimilar forms of periodization on insulin resistance indicators and found no differences [15, 18]. Strohacker et al. (2015) understood that it is premature to conclude that periodized training is superior to non-periodized training in terms of improving health indicators in non-athletes [38]. However, they stated the need for more research to understand the effectiveness of periodization and the feasibility of implementing flexible methods. To date, an insufficient number of studies have investigated this topic.

Inoue et al. (2015) built interdisciplinary therapy models that included periodized combined training (linear versus undulating) and realized that both ways were effective in improving the lipid profile and insulin sensitivity in adults with obesity [18]. Still, with this population, and with similar comparisons, Foschini et al. (2010) demonstrated a reduction in insulin and HOMA-IR concentrations only in the group that used daily undulating periodization [39]. An important detail is that the participants in this prior study [39] and ours were not previously trained, which results in highly variable responses independent of the periodization model. However, such findings reinforce the relevance of structured physical exercise in modulating these metabolic variables regardless of the periodization. For inactive and/or unfamiliar people with physical exercise, the type of periodization does not seem to interfere with the effects of glucose metabolism in the first months of training. In this sense, gaps are indicated in the literature about the superiority of periodized training in health outcomes in special populations. Clinically, the results of periodization are relevant, as they suggest significant reductions in insulin resistance indicators in a population at metabolic risk. In general, these positive effects can be effective in the prevention and treatment of obesity, as well as T2DM and other diseases, by promoting adjustments in adipocytokines and other metabolic markers [40].

The strengths of this blind randomized clinical trial were the control of the aerobic training variables, the maintenance of the relative intensity in the NG, and the gradual increase of intensity in the PG over the mesocycles, both adjusted by the resting heart rate and considered a control method of low-cost. The progression of intensity and similarity in the training volume, is another strength, as it allows to verify the effects of periodization in the combined training program in an equal way. Something no less important to be considered is that, even with low training intensity, as well as low weekly frequency, there were still improvements in glycemic metabolism markers in this population sample. These are important findings—particularly for a group that often has difficulty starting and maintaining a regular exercise program [41]. Nevertheless, the results presented here indicate moderate magnitude reductions, based on effect size and the individual responsiveness of the participants, for insulin resistance indicators of both training groups. Considering the specificity of the population, as well as the relatively low volume of training proposed, this is a satisfactory and clinically relevant result.

However, limitations, such as the low frequency of participants in the training program and sample loss, are also recognized due to the lack of data on the outcome variables of this study, which can lead to a low sampling power for statistically significant findings. Besides, the lack of control of food intake is an important consideration, as there are significant associations between dietary patterns and insulin resistance [42]. The lack of glycated hemoglobin may be another limitation. Whereas the fasting blood glucose test measures the blood glucose concentration only at the time of collection, while the glycated hemoglobin test can show an average of blood glucose concentrations in the last 3 months, being considered a better predictor of risk cardiometabolic [43]. Also, it is plausible to consider the biological individuality of the participants, their respective routines, and other factors that could not be measured here as determinants in the changes, whether they were highlighted or not. Other studies should investigate such outcomes in other age groups, with a higher percentage of adherence to training sessions and other forms of periodization, which can influence the results that will be found.

## Conclusion

Thus, 16 weeks of periodized and non-periodized combined training similarly decreases insulin and HOMA-IR levels in adults with obesity. The training used, which has practical applicability, even with low adherence, provided a reduction in important risk factors for triggering other comorbidities in a population already considered at risk. For future studies, greater attention is recommended to these health indicators in adults with obesity not yet diagnosed with other comorbidities. In addition, we recommended the implementation of adherence strategies to enhance the results of this study, as well as further exploration of other training methods.

## Data Availability

The datasets used and/or analyzed during the current study are available from the corresponding author on reasonable request.

## Abbreviations

T2DM: Type 2 diabetes mellitus
BMI: Body mass index
CG: Control group
NG: Non-periodized training group
PG: Linear periodization group
%HRres: Reserve heart rate ranges
GEE: Generalized estimated equation
MR: Maximum repetitions
